# The Effect of Density-Dependent Phase on the Locust Gut Bacterial Composition

**DOI:** 10.3389/fmicb.2018.03020

**Published:** 2019-01-18

**Authors:** Omer Lavy, Uri Gophna, Eran Gefen, Amir Ayali

**Affiliations:** ^1^School of Zoology, Tel Aviv University, Tel Aviv, Israel; ^2^School of Molecular Cell Biology & Biotechnology, Tel Aviv University, Tel Aviv, Israel; ^3^Department of Biology, University of Haifa – Oranim, Kiryat Tivon, Israel

**Keywords:** insect – symbiont interaction, bacterial community, endosymbiont, *Enterobacteriaceae*, locust microbiota, gut bacteria

## Abstract

The desert locust demonstrates density-dependent phase polyphenism: For extended periods it appears in a non-aggregating, non-migrating phenotype, known as the solitary phase. When circumstances change, solitary individuals may aggregate and transform to the gregarious phenotype, which have a strong propensity for generating large swarms. Previous reports have suggested a role for gut-bacteria derived volatiles in the swarming phenomenon, and suggested that locusts are capable of manipulating their gut microbiome according to their density-dependent phases. Here, we directly tested this hypothesis for the first time. Using locusts of both phases from well-controlled laboratory cultures as well as gregarious field-collected individuals; and high-throughput sequencing. We characterized the hindgut bacterial community composition in the two phases of the desert locust. Our findings demonstrate that laboratory-reared gregarious and solitary locusts maintain a stable core of *Enterobacter*. However, while different generations of gregarious locust experience shifts in their *Enterobacter*’s relative abundance; the solitary locusts maintain a stable gut microbiome, highly similar to that of the field-collected locusts. Tentative phase differences in wild populations’ microbiome may thus be an indirect effect of environmental or other factors that push the swarming individuals to homogenous gut bacteria. We therefore conclude that there are phase-related differences in the population dynamics of the locust hindgut bacterial composition, but there is no intrinsic density-dependent mechanism directly affecting the gut microbiome.

## Introduction

Insect–bacteria symbiotic interaction is a common phenomenon, described in several insect orders (Moran and Baumann, 2000; Oliver et al., 2010; Bennett and Moran, 2013; Salem et al., 2015). Various aspects of these insect–host interactions have been reported, including the production of bacteria-derived nutrients (Feldhaar et al., 2007; Salem et al., 2014), enhancement of the insect stress and pathogen resistance (Kaltenpoth, 2009; Oliver et al., 2010), production of bacteria-derived insect aggregation-inducing agents (Dillon et al., 2000; Wada-Katsumata et al., 2015), and more.

One studied example of insect–bacteria interactions is that of locusts and grasshoppers. The desert locust (*Schistocerca gregaria* Forskål) is a swarm-forming species, known since ancient times to cause massive damage to crops. Desert locust swarms originating in Africa can reach the Middle East, India, and southern Europe (Pener and Simpson, 2009; Cullen et al., 2017; Food and Agriculture Organization [FAO], 2017), destroying cultivated fields and local vegetation, with a devastating impact on farmers’ livelihood (Food and Agriculture Organization [FAO], 2017). Like all locust species, *S. gregaria* demonstrates two very different density-dependent phases: solitary and gregarious. The two phenotypes differ in their morphology, behavior, and physiological traits (Pener and Simpson, 2009; Cullen et al., 2017).

Research of the locust microbiome has focused to date on gut microbiota. The gut bacterial community composition of adult *S. gregaria* has been thoroughly characterized using bacteria culturing and in some cases denaturing gradient gel electrophoresis (DGGE) (Dillon and Charnley, 2002; Dillon et al., 2005, 2010). Those studies revealed the locust hindgut as an important stable niche suitable for bacterial establishment, and as a crucial site in the bacteria–host interaction (Dillon and Charnley, 2002). The locust hindgut was found to be dominated by Gammaproteobacteria members, mostly of the family *Enterobacteriaceae* and the Firmicutes family *Enterobacteriaceae* (Dillon and Charnley, 2002). Although locusts have not been shown to develop an apparent obligatory relationship with certain bacterial species, they do seem to have close and constant interactions with specific *Enterobacteriaceae* representatives, such as *Pantoea agglomerans, Klebsiella pneumoniae*, and *Enterobacter cloacae* (Dillon and Charnley, 2002; Dillon et al., 2005, 2010). To the best of our knowledge, practically all studies to date focused on mature gregarious individuals only.

A healthy diverse hindgut bacterial community contributes to the locust’s resistance to pathogens by way of colonization-resistance and phenolic compound secretion (Dillon and Charnley, 1995, 2002). Some of these bacteria-derived phenolic volatile molecules (Phenol and Guaiacol) were found to be electrophysiologically active compounds that function as aggregating agents (reviewed in Pener and Simpson, 2009), suggesting a bacterial role in swarm gregariousness preservation (Dillon et al., 2000, 2002). Such a putative role of gut bacteria in aggregation behavior may suggest, in turn, phase-related differences in the locust gut microbiome. Dillon et al. (2008) compared, for the first time, the bacterial community composition of solitary and gregarious individuals. These were collected from field populations of the brown locust (Orthoptera: *Locusta Paradalina*). Using DGGE and 16S rRNA sequence analysis, those authors observed that the solitary individuals harbored a simpler hindgut bacterial community in comparison with their gregarious conspecifics. They also suggested that these phase-dependent bacterial diversity-related differences may act to augment the gregarious superior pathogen resistance, as found by Wilson et al. (2002).

Nevertheless, a controlled laboratory comparative study of the microbiome of the two phases of major swarm-forming locust species, including *S. gregraia*, has not been performed to date. Such a study can be greatly assisted by the power of next generation sequencing methods, which are now becoming increasingly accessible. Finding a “gregarious/solitary bacterial signature” could provide important general insights into the interactions of the organism, its environment (in this case rearing density), and the microbiome. Any differentially expressed bacteria could of course also have the potential to impact locust phase-related behavior and physiology, and potentially be harnessed to the efforts of controlling locust outbreaks.

## Materials and Methods

### Laboratory-Reared Locusts

Locusts were kept in a temperature-controlled room at 30°C under a 10D:14L photoperiod cycle, with electric bulbs providing a radiant heat source during daytime (bringing daytime temperature up to ∼37°C), allowing behavioral thermoregulation. Gregarious locusts were reared for many consecutive generations under heavy crowding of 300–500 individuals in 140 l wooden cages. In order to obtain locusts in the solitary phase, hatchlings from eggs laid by gregarious females were collected within 3 h of hatching and reared in isolation until adulthood (Geva et al., 2010; Berman et al., 2013). All animals were fed daily with fresh wheat seedlings and dry oats. Animals of both phases were reared in separate rooms, under similar ambient conditions except for density. Gregarious and solitary locusts were sampled simultaneously (individuals of the same generation) in May 2016, February 2017, and July 2018.

### Field-Collected Locusts

Field gregarious Locusts were collected in Israel at the Kmehin area (30.920N/34.4275E) during a rare swarming-locust infestation on March 2013. The specimens were kept at -20°C until further use.

### Hindgut Sampling

Locust’s wings and limbs were initially removed. Each individual was then submerged for 2 min in a 1% NaOCl solution for surface sterilization, and then washed twice with filtered, double-distilled water. The insects were dissected aseptically under filtered saline solution (0.15 M NaCl). 3% NaOCl and fire were used for sterilization of the working station and dissection tools, respectively. Excised hindgut samples were kept individually in 70% absolute ethanol at -20^o^C until further use.

### DNA Extraction and Sequencing

Ethanol was removed and bacterial genomic DNA was extracted using the “Powersoil” DNA isolation Kit (Mo Bio Laboratories Inc., Carlsbad CA, United States), according to the manufacturer’s instructions, using 60 μl for final DNA elution.

To determine bacterial composition, polymerase chain reaction (PCR) of variable areas V3 and V4 of the prokaryotic 16S rRNA gene was applied on the extracted DNA; using a universal primers containing 5-end common sequences (CS1-341F 5′-ACACTGACGACATGGTTCTACANNNNCCTACGGGAGGC AGCAG and CS2-806R 5′-TACGGTAGCAGAGACTTGG TCTGGACTACHVGGGTW TCTAAT).

Thirty-one PCR cycles (95°C 15 s, 55°C 15 s, 72°C 5 s) were performed using a PCR master mix KAPA2G Fast^TM^ (KAPA Biosystems, Wilmington, MA, United States). PCR product validation was conducted by agarose gel 1% electrophoresis.

Deep sequencing of the amplified amplicons was conducted on an Illumina MiSeq platform at the Chicago Sequencing Center of the University of Illinois. To ensure data evenness, before analysis the data were rarefied to 10,000 seqs/sample.

### Data Analysis

Demultiplexed raw sequences were quality filtered (bases with a PHRED score < 20 were removed) and merged using PEAR (Zhang et al., 2014). Sequences of less than 380 bp (after merging and trimming) were discarded. Data were then analyzed using the Quantitative Insights Into Microbial Ecology (QIIME) package (Caporaso et al., 2010). Vsearch (Rognes et al., 2016) was used for chimera detection and elimination; OTU picking (0.99 similarity) and taxonomy assignment were done using Silva database (version 128). After filtering, samples contained 10,120–54,264 reads per sample. Shannon biodiversity index for the different samples and Flinger-Killeen test of homogeneity of variances for those diversity values were calculated with “R” v.3.4.1. (R Core Team, 2013). Analysis of similarities-“Anosim,” principal coordinate analysis (PCoA) and canonical analysis of principal coordinates (field locusts were treated as a third phase-group) were carried out using the “vegan 2.4-3” package (Oksanen et al., 2008).

Linear discriminant analysis with effect size estimation (LEfSe) (Segata et al., 2011) was used to analyze genus level OTU tables applying the online analysis tool available from http://huttenhower.sph.harvard.edu/galaxy/. The LEfSe algorithm allows the identification of the most significant taxa, differentiating between the gregarious and solitary group, considering a *p*-value < 0.05 as significant. The LEfSe significant results were corrected to prevent false discovery rate (FDR) using “R” v.3.4.1. (R Core Team, 2013).

## Results

We successfully sequenced the hindgut bacterial composition of 28 laboratory-reared gregarious males (referred to hereafter as gregarious locusts), 20 laboratory-reared solitary males and 5 field-collected gregarious males (referred to hereafter as field locusts; SRA archive accession number: PRJNA503121).

**FIGURE 1:**
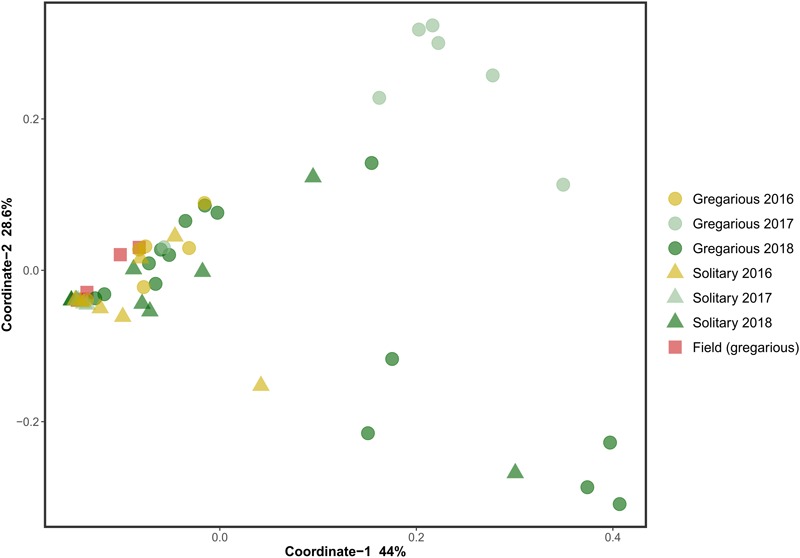
Weighted UniFraq principal coordinate analysis (PCoA) of locust hindgut bacterial composition.

Solitary and field locust gut microbiota, was dominated by bacteria of the phylum Proteobacteria across samples in all 3 years. In contrast, the most abundant phylum in the gregarious locusts changed from one sampling to the next. In the 2016 samples, Proteobacteria members dominated the gut bacterial community, in 2017 samples, Firmicutes were the most abundant, and Proteobacteria became most abundant again during the 2018 sampling, along with Cyanobacteria that are probably food-derived (Supplementary Figure S1). Unweighted UniFraq principal coordinate analysis (PCoA) did not suggest any phase-related differentiation (Supplementary Figure S2), whereas weighted UniFraq PCoA indicated variation among gregarious groups across different years (Figure 1).

This trend of composition shifts was rooted at the genus level. Analysis of similarities (ANOSIM) for the genus-level bacterial composition of all gregarious (*N* = 28) and solitary individuals (*N* = 20) showed a significant difference in the gut bacterial composition between the two phases (*p* < 0.001, *R* = 0.3). Furthermore, while the gregarious gut bacterial composition of different sampling years demonstrated significant inconsistency (gregarious 3 years ANOSIM comparison, *p* < 0.001, *R* = 0.35; gregarious pair-wise year ANOSIM comparison: 2016–2017, *p* = 0.002, *R* = 0.74; 2016–2018, *p* = 0.038, *R* = 0.214; 2017–2018, *p* = 0.002, *R* = 0.34), the solitary locust composition remained similar across the three sampling seasons (solitary 3 years ANOSIM comparison, *p* = 0.17, *R* = 0.07). In addition, the solitary group showed no significant difference in microbiome composition when compared to the field locust (ANOSIM, *p* = 0.5, *R* = -0.02).

**FIGURE 2:**
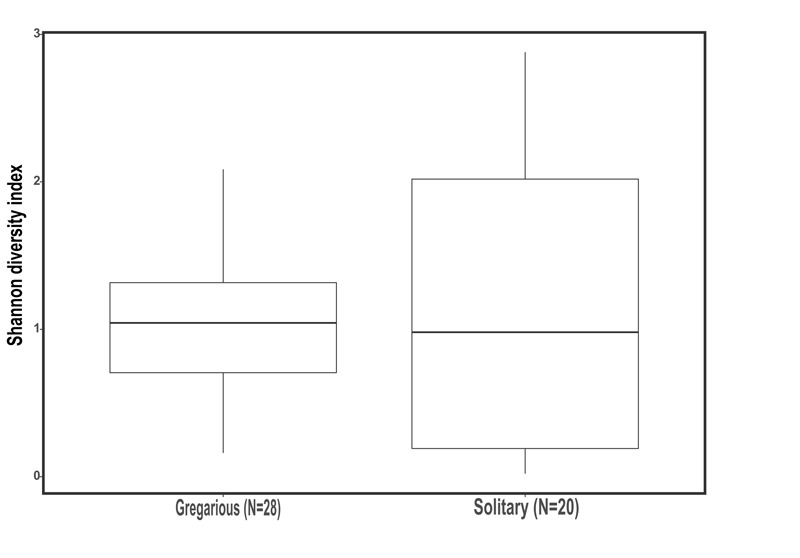
Shannon biodiversity index of gregarious and solitary locusts’ hindgut bacterial community composition (Mann Whitney- *U* test *p* = 0.94).

Genus biodiversity means of the two laboratory-reared locust phases were broadly similar, with a Shannon biodiversity-index of 1.06 (*SD* = 0.54) for the gregarious hindgut community composition and 1.16 (*SD* = 0.97) for the solitary composition (Figure 2). Flinger-Killeen test of homogeneity of variances for the Shannon diversity indices, showed within-phase homogeneity and between-phase heterogeneity (Table 1).

**Table 1:** Tests for homogeneity of variances.

Groups compared	Chi-squared	df	*p*-value
Gregarious- between years comparison	0.74	2	0.69
Solitary- between years comparison	4.3	2	0.11
Solitary Vs. Gregarious	10.28	1	0.001

*Within-phase and between-phase comparisons using the Flinger-Killeen test (the null hypothesis is that the variances in each of the groups are the same).*

**FIGURE 3:**
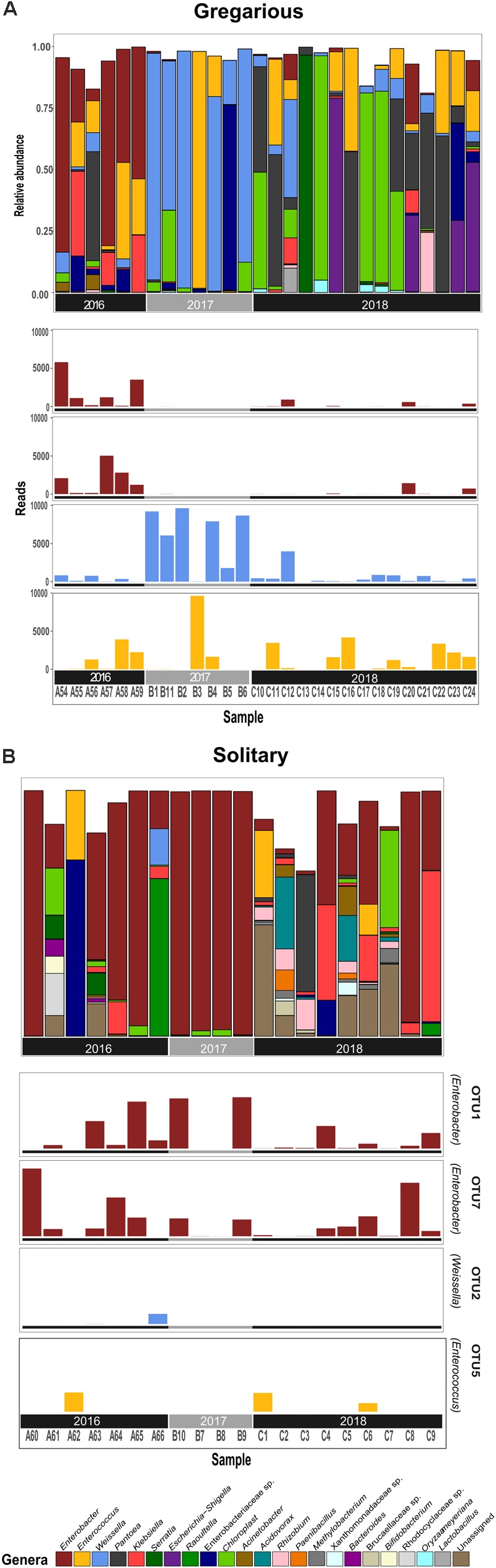
Gregarious **(A)** and Solitary **(B)** per sample relative abundance of genera consisting at least 5% of an individual’s bacterial composition, with the phase-differentiating genera’s most abundant OTU’s.

**FIGURE 4:**
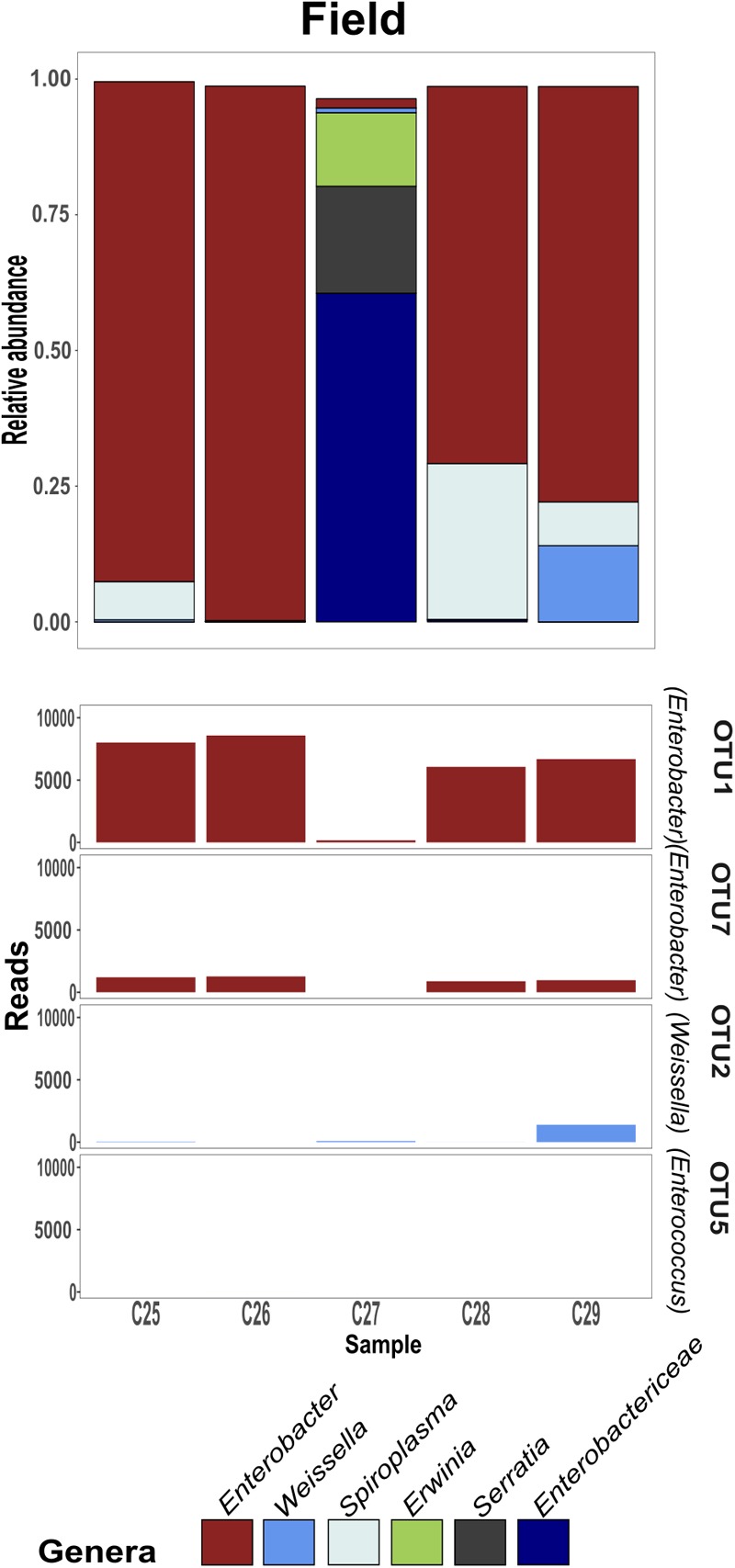
Field-collected gregarious per sample relative abundance of genera consisting at least 5% of an individual’s bacterial composition, with the phase-differentiating genera’s most abundant OTU’s.

**FIGURE 5:**
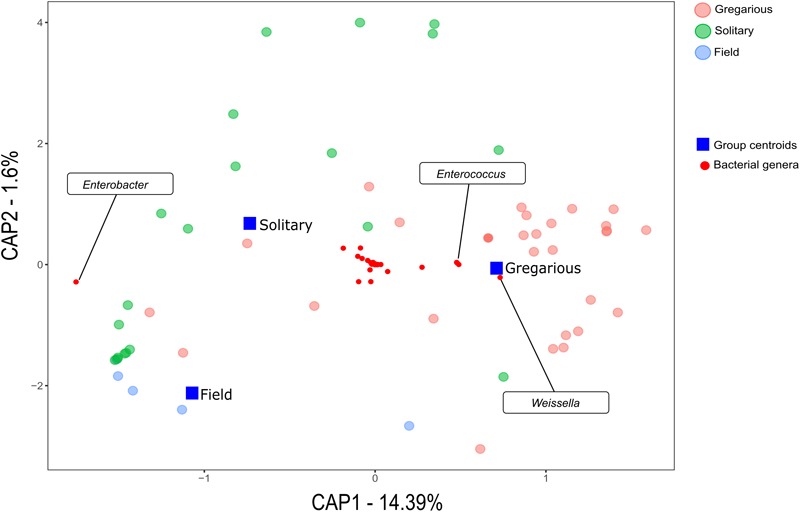
Genus phase-constrained canonical analysis of principal coordinates.

Linear discriminant analysis with effect size estimation analysis (Segata et al., 2011) indicated the *Enterobacter* (Proteobacteria), *Enterococcus* (Firmicutes), and *Weissella* (Firmicutes) genera as differing in relative abundance between gregarious and solitary samples (FDR-adjusted *p*-values for all three genera: *p* < 0.001). The genus *Enterobacter* remained relatively constant in its high relative abundance and was the dominant bacteria in the solitary and field locust samples (Figures 3, 4). Phase-constrained canonical analysis of principal coordinates, confirms the dominance of *Enterobacter* among field and solitary locusts, whereas *Enterococcus* and *Weissella* are more gregarious-associated (Figure 5).

**FIGURE 6:**
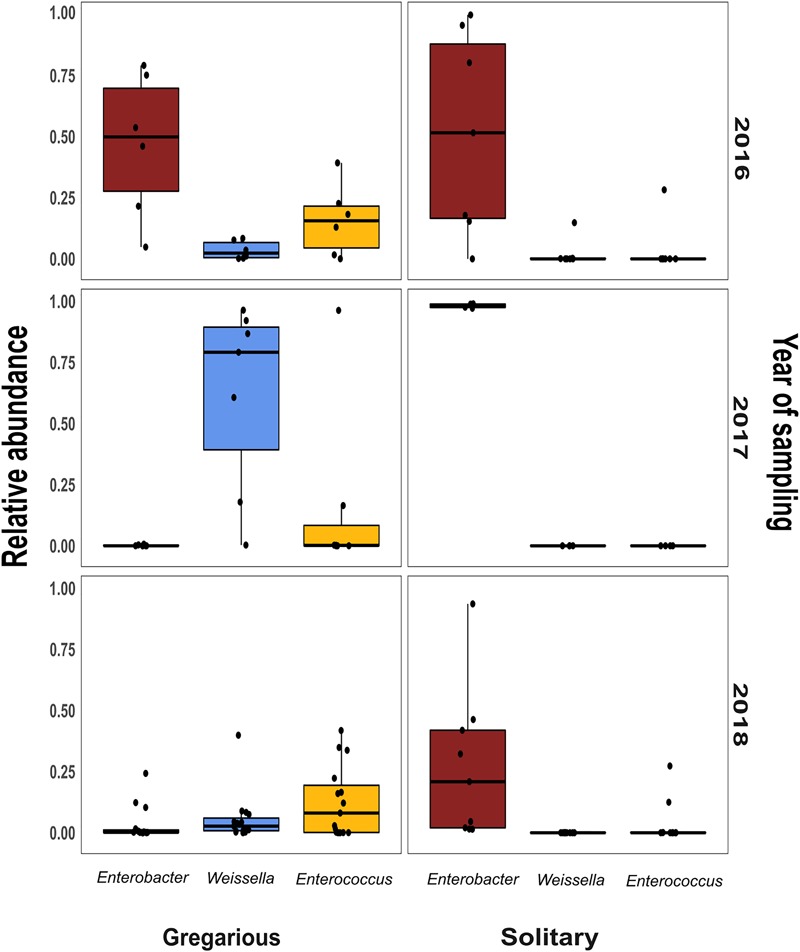
Per year relative abundance of the three phase-differentiating bacterial genera, in the gregarious and solitary locusts.

While the gregarious locusts of 2016 were also dominated by *Enterobacter*, in 2017 and 2018 gregarious animals, the *Enterococcus* and *Weissella* genera were dominant, along with other less consistent bacterial genera (Figures 3, 6). The majority of *Enterobacter* 16S rRNA gene sequences grouped into two Operational Taxonomic Units (OTUs), denoted “OTU1” and “OTU7” that were widely common, and shared among the solitary samples, the field locust samples and the 2016 gregarious samples (Figures 3, 4). The sequences taxonomically assigned to *Weissella* clustered mainly under “OTU2” that was very prevalent in the gregarious samples and less so in the solitary and field samples. The main *Enterococcus* OTU was “OTU5” that showed the same overall pattern of abundance as “OTU2” (Figure 3). All four OTU’s reads-count followed their respective genera’s relative abundance pattern, suggesting a specific bacterial species as the cause of shifts in gregarious hindgut bacterial composition.

## Discussion

Previous findings have suggested that *S. gregaria* hindgut bacterial community composition is quite simple, consistent, and dominated by *Enterobacteriaceae* members (Dillon and Charnley, 2002). Our current results partly support these findings, and highlight an especially strong association with the genus *Enterobacter*. Though its relative abundance in the gregarious population tend to dynamically shift (Figure 3), its constant presence in the solitary individuals and the sharing of dominant OTU’s between the laboratory stock and the field locusts (Figures 3–5) indicate that *S. gregaria* maintain some core *Enterobacter* population, and imply *trans-* generational inoculation of *Enterobacter* members.

Bacterial symbionts in the locust hindgut were also suggested to play a role in locust swarming behavior (Dillon et al., 2000, 2002). However, the observation of bacterial composition shifts among the gregarious locust (Figures 1, 3) may seem inconsistent with the role attributed to these bacteria in releasing conspecific cohesion volatiles. A possible reason for the herein reported gregarious bacterial inconsistency may lie with the *Weissella* and *Enterococcus* genera that became the dominant bacterial genera in the gregarious locusts in 2017, and remained dominant alongside a variety of *Enterobacteriaceae* members in the 2018 samples (Figure 3). As noted, some *Enterococcus* species isolated from *S. gregaria* were reported to produce small quantities of guaiacol (Dillon and Charnley, 2002). Wada-Katsumata et al. (2015) observed that the presence of *Weissella* and *Enterococcus* in feces of the German cockroach (*Blattella germanica*), facilitates nymph aggregation, suggesting the possibility that these bacteria, compensate for the loss of *Enterobacter* -derived volatiles.

The microsporidian pathogen *Paranosema (Nosema) locustae*, was found to alter the acidification level in the migratory locust (*Locusta migratoria*) hindgut, thus inhibiting the growth of *Enterobacter* while enabling the reproduction of *Weissella* and *Enterococcus* isolates (Shi et al., 2014). Though we did not test for *P. locustae* presence, this may be a cause for changes in the gregarious locust bacterial composition. If so, it indicates that the high density of the swarm, is a very efficient force, spreading microorganisms from one individual to another, unifying their microbiome. This is supported by the relatively low standard deviation and high homogeneity of the gregarious biodiversity values in comparison with the values observed for solitary locusts (Figure 2 and Table 1). The presence of *Weissella* and *Enterococcus* of the same OTU’s at very low frequencies in the solitary insects (Figure 3) further supports this notion since solitary rearing prevented spreading from infected to non-infected individuals.

Our results of the solitary locust microbiome are also somewhat inconsistent with the idea of a *Enterobacteriaceae* species-related gregarization factor. However, within the context of the low density solitary-phase, concentration of emitted bacterial volatiles may not be sufficient to facilitate aggregation. This is in accord with the findings of Njagi et al. (1996), who demonstrated traces of phenole and guaiacol emitted from both gregarious and solitary individuals, and furthermore recorded a similar EAG (electroantennography) response from males and females of both phases when exposed to these volatiles.

When sampling a wild mixed-phase population of the brown locust, Dillon et al. (2008) reported a microbiome phase-dependent difference. The authors concluded that since the gregarious and solitary individuals occupied the same habitat, the different bacterial composition was due to an intrinsic phase-related ability to regulate the gut bacterial community composition. According to the hypothesis raised by Dillon et al. (2005), a high bacterial diversity in the gregarious hindgut might entail a high energetic cost, but it benefits the host by providing better pathogen resistance, which in turn may be critical in the high density of the swarm.

The current study is the first attempt to directly compare the bacterial composition of the two locust phases in laboratory-reared insects. The controlled conditions are important as they enabled us to attribute any tentative difference found to specific aspects of the phase phenomenon, specifically to the rearing density (in contrast to other factors such as food plants or other aspects of the locust’s micro-environment and behavior). The surprising high resemblance of the solitary hindgut bacterial composition with that of the field locust, serves as a strong evidence for the relevance of the results to understanding of locust–bacteria interactions in the wild. Our findings suggest not only that gregarious individuals do not harbor a higher diversity of gut bacteria (Figure 2), but that as a group they harbor even fewer bacterial species than their solitary conspecifics, probably due to their high density.

The fact that the bacterial microbiota of gregarious desert locust significantly changed over time, while the microbiota of their solitary conspecific remained relatively constant (when fed the same diet and kept under similar overall conditions, except density), indicates that, at least in this species, there is no density-dependent intrinsic mechanism controlling bacterial community composition. It is here suggested that the locust swarming behavior indirectly acts as a unifying force, pushing the microbiome of gregarious individuals toward similar bacterial composition, while maintaining core *Enterobacter* populations in both gregarious and solitary locusts.

## Author Contributions

AA, OL, and EG conceived the study and designed the experiments. OL conducted the experiments, analyzed the data, and wrote the manuscript. UG oversaw microbial ecology experiments and guided data analysis. AA, UG, and EG commented on and revised the manuscript.

## Conflict of Interest Statement

The authors declare that the research was conducted in the absence of any commercial or financial relationships that could be construed as a potential conflict of interest.

## Supplementary Material

FIGURE S1 Per-sample relative abundance of phyla for gregarious **(A)**, solitary **(B)** and field- collected **(C)** locusts.

FIGURE S2 Unweighted UniFraq principal coordinate analysis (PCoA) of locust hindgut bacterial composition.
