# Supplementary figures and images for: The Effect of Density-Dependent Phase on the Locust Gut Bacterial Composition
Source: Front Microbiol. 2019 Jan 18;9:3020. doi: 10.3389/fmicb.2018.03020 (PMC6345702; doi:10.3389/fmicb.2018.03020)

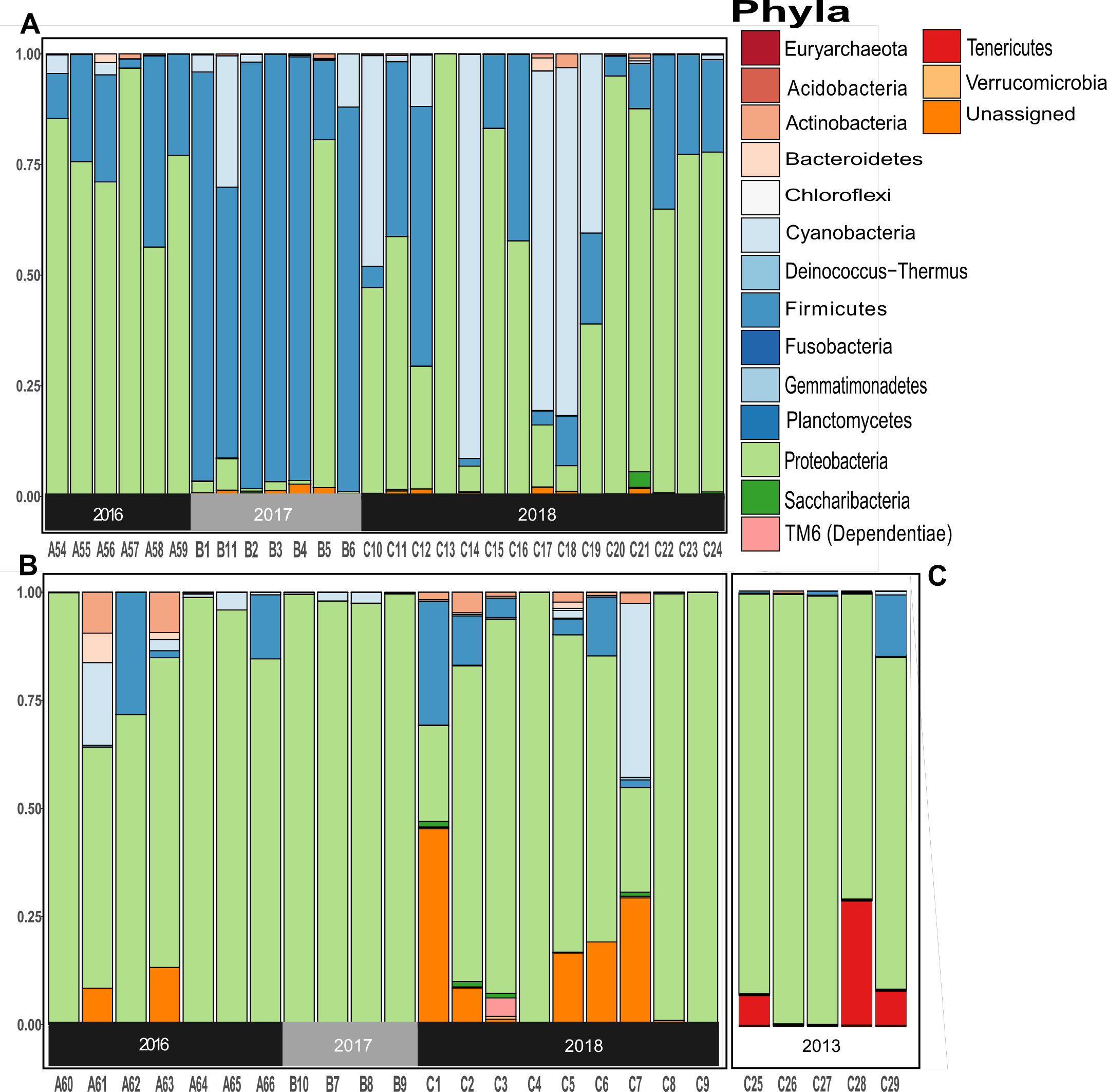

Supplement: FIGURE S1 — Per-sample relative abundance of phyla for gregarious (A), solitary (B) and field- collected (C) locusts. [file Image_1.TIFF]

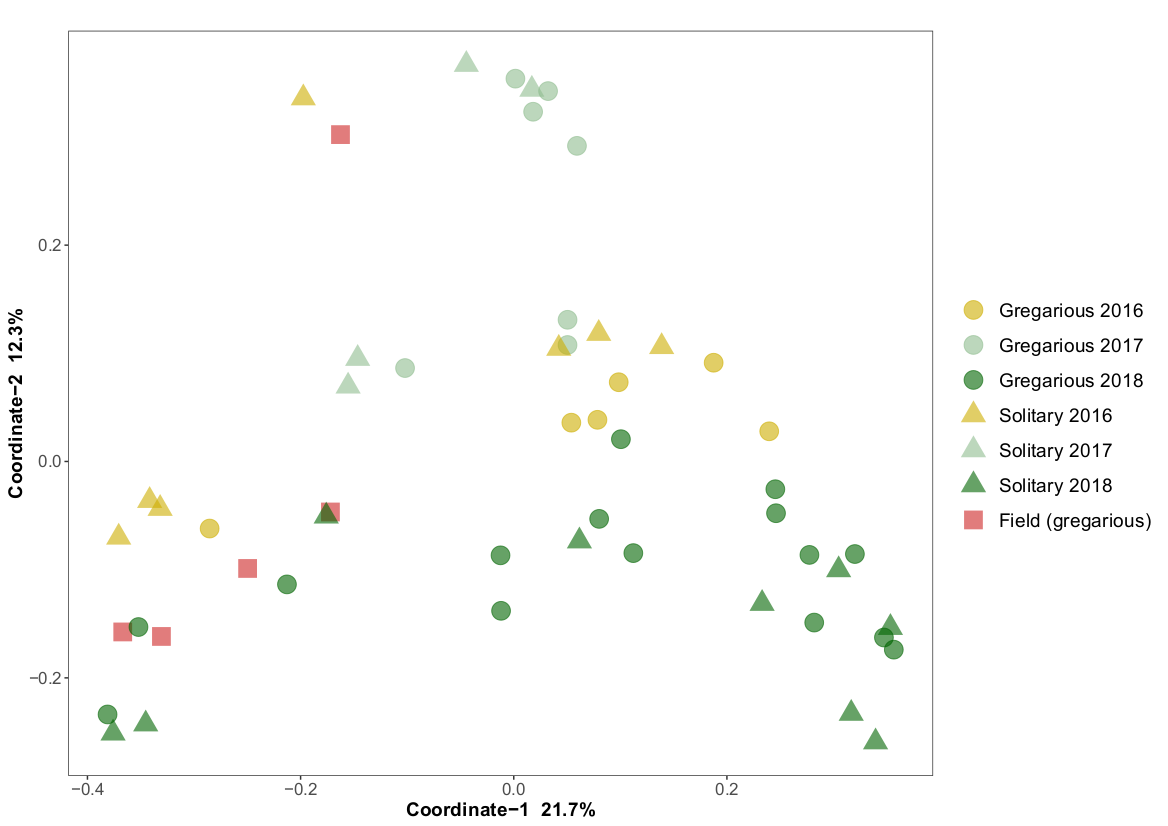

Supplement: FIGURE S2 — Unweighted UniFraq principal coordinate analysis (PCoA) of locust hindgut bacterial composition. [file Image_2.TIFF]
